# Probing *Synechocystis*-Arsenic Interactions through Extracellular Nanowires

**DOI:** 10.3389/fmicb.2016.01134

**Published:** 2016-07-19

**Authors:** Sandeep Sure, M. L. Ackland, Aditya Gaur, Priyanka Gupta, Alok Adholeya, Mandira Kochar

**Affiliations:** ^1^TERI-Deakin Nano biotechnology Centre, The Energy and Resources InstituteGurgaon, India; ^2^Centre for Cellular & Molecular Biology, Deakin University, MelbourneVIC, Australia

**Keywords:** microbial nanowires, arsenic, pili, *Synechocystis*, PilA

## Abstract

Microbial nanowires (MNWs) can play an important role in the transformation and mobility of toxic metals/metalloids in environment. The potential role of MNWs in cell-arsenic (As) interactions has not been reported in microorganisms and thus we explored this interaction using *Synechocystis* PCC 6803 as a model system. The effect of half maximal inhibitory concentration (IC_50_) [~300 mM As (V) and ~4 mM As (III)] and non-inhibitory [4X lower than IC_50_, i.e., 75 mM As (V) and 1 mM As (III)] of As was studied on *Synechocystis* cells in relation to its effect on Chlorophyll (Chl) a, type IV pili (TFP)-As interaction and intracellular/extracellular presence of As. *In silico* analysis showed that subunit PilA1 of electrically conductive TFP, i.e., microbial nanowires of *Synechocystis* have putative binding sites for As. In agreement with *in silico* analysis, transmission electron microscopy analysis showed that As was deposited on *Synechocystis* nanowires at all tested concentrations. The potential of *Synechocystis* nanowires to immobilize As can be further enhanced and evaluated on a large scale and thus can be applied for bioremediation studies.

## Introduction

Arsenic (As) is one of the most widely distributed toxic elements on earth and has become a major cause of concern in recent years due to increased human activities like mining, well-drilling, pesticide use, burning of fossil fuels, etc. (Yin et al., 2011; Shen et al., 2013). Inorganic As is mainly found in two common oxidation states, i.e., trivalent arsenite [As (III)] and pentavalent arsenate [As (V)]. As (III) is not only more toxic, but also shows greater solubility (4–10 times) than As (V). As (III) exerts its toxic effect by binding with -SH (sulfhydryl) sites of various proteins while As (V) inhibits oxidative- as well as photo-phosporylation (Miyashita et al., 2012).

In the environment, As concentrations varies from 5 mg L^-1^ (in earth’s crust) to as high as 130,000 mg L^-1^ (in acid mine water; Shen et al., 2013; Majzlan et al., 2014). Microorganisms are known to play an important role in cycling of As in the environment (Huang et al., 2014). Cell-As interactions have been widely studied in different cyanobacteria (Huang et al., 2014) and *Synechocystis* PCC 6803 is known to have an intricate machinery involving enzymes [e.g., As (V) reductase, methylase, etc.], transporters etc. to interact with As (Lopez-Maury et al., 2003). The As resistance operon (*arsB*, *arsC*, and *arsH*) in *Synechocystis* is known to be induced in the presence of As (III) and antimony (III), but not As (V) (Lopez-Maury et al., 2003). ArsB is involved in extrusion of As (III) while ArsC is thought to play a role in As (V) detoxification (Lopez-Maury et al., 2003). ArsH has been shown to have a quinone reductase activity and hypothesized to protect the cells from oxidative stress caused by As (III) (Hervaìs et al., 2012).

Apart from ArsC, two secondary As (V) reductases (ArsI1 and ArsI2) have also been observed in *Synechocystis* (López-Maury et al., 2009). *Synechocystis* produces *S*-adenosyl methionine methyltransferase (ArsM) which methylates As (III) to the almost non-toxic methylated species (Yin et al., 2011). Additionally, *Synechocystis* has been shown to produce arsenosugars from inorganic As (Miyashita et al., 2012). However, organic As species do not seem to be part of the detoxification mechanisms and may be formed for different purpose (e.g., membrane lipid bilayers; Miyashita et al., 2012). The conversion of As (V) to As (III) and its subsequent efflux from the cells is one of the major As detoxification mechanisms in *Synechocystis* (Zhang et al., 2013; Sánchez-Riego et al., 2014). This sophisticated machinery may give *Synechocystis* an ability to tolerate relatively high levels of As compared to other cyanobacteria (Wang et al., 2013; Huang et al., 2014) and also implies that it might be playing an important role in As biogeochemistry (Yin et al., 2011). Research on *Synechocystis*-As interactions has considered it as a toxic molecule at specific concentrations and describes mechanisms that deal with As toxicity. However, growing number of studies in various bacteria and cyanobacteria suggests its use as a bioenergetic molecule (Kulp et al., 2008; van Lis et al., 2013; Nagy et al., 2014). Further, in various bacteria, extracellular structures like pili, extracellular polysaccharides, outer membrane vesicles, etc. have been shown to play a role in cell-metal interaction (Reguera et al., 2005; Gorby et al., 2008; Cologgi et al., 2011; Planchon et al., 2013).

In this study, we investigated the effect of As on growth behavior of *Synechocystis* cells. Type IV pili (TFP) in *Synechocystis* are electrically conductive in nature, i.e., they can act as microbial nanowires (Sure et al., 2015). The influence of As on the production of TFP in *Synechocystis* and their potential role in *Synechocystis*-As interactions has not been explored so far. This study describes the intracellular and extracellular morphological changes induced by As and indicates a potential role of *Synechocystis* TFP in cell-As interactions.

## Materials and Methods

### Growth Conditions

*Synechocystis* PCC 6803 was cultured and maintained in BG11 medium (Stanier et al., 1971) supplemented with 0.05 mM NaHCO_3_. The culture was grown in non-shaking (static) conditions, at 25–27°C and under alternate light/dark (16 h/8 h) conditions with light intensities of ~40–50 μmol photons m^-2^ s^-1^. To study the involvement of iron (Fe) and manganese (Mn) in extracellular As precipitation, the *Synechocystis* cells were also grown in Fe^-^Mn^-^ BG11, i.e., BG11 medium lacking Fe (Ammonium ferric citrate) and Mn (Manganese chloride) sources. For As studies, sodium arsenate (Na_2_HAsO_4_.7H_2_O), i.e., As (V) (Loba Chemie, India) and sodium arsenite (NaAsO_2_), i.e., As (III) (Loba Chemie, India) were used while for sodium ion (Na^+^) studies, sodium chloride (NaCl) (Fisher Scientific) was used. Ten% inoculum of OD_750_ 1.2 from exponential growth phase cultures were used for all experiments which were done in triplicate. *Synechocystis* cells studied here were grown and maintained in As free conditions for over 3 years in our lab.

### Determination of Chlorophyll-a Concentration of *Synechocystis* Cells

The chlorophyll (Chl) a concentration of *Synechocystis* cells was determined as described previously (Wintermans and De Mots, 1965; Knudson et al., 1977; Luo et al., 2013; Wang et al., 2013; Gupta and Ballal, 2015) with some modifications. Briefly, 1 ml cell culture was centrifuged at 6000 rpm for 4 min. After centrifugation, 950 μl supernatant was removed and an equivalent amount of 100% ethanol was added to the cell pellet which resulted in a final concentration of 95% ethanol. The pellet was fully resuspended in ethanol by vortex mixing. This solution was kept at 4°C for 24 h. After 24 h, the cells were centrifuged again and supernatant was collected in a fresh vial. The absorbance of resultant supernatant was taken at 665 nm and the Chl a was calculated using the following formula;

$$Chl\,\;\;\;a\left(mg/L\right)=13.70\times A_{665\,\;\;nm.}$$

### Determination of Half Maximal Inhibitory Concentration (IC_50_) of as for *Synechocystis* Cells

Preliminary screening of *Synechocystis* cells for tolerance to As was carried out [from 4 to 6 mM As (III) and from 300 to 400 mM As (V)]. After preliminary studies, Chl a estimation was used in conjunction with measurement of change in optical density (OD_750_) to study the inhibitory effect of As on *Synechocystis* cells. Sodium arsenate contains Na^+^which can inhibit cell growth at higher concentrations. Thus to study the effect of Na^+^ on the cell growth, cells were treated with 600 mM of NaCl [i.e., the amount of Na^+^ present in 300 mM of sodium arsenate (Na_2_HAsO_4_.7H_2_O)] and their growth was monitored along with As (V) treated and control cells. *Synechocystis* cells were also grown with non-inhibitory concentrations of As [4X lower than IC_50_, i.e., 75 mM As (V) and 1 mM As (III)] in BG11 broth and its growth was monitored by Chl a and OD_750_ measurement.

### Microscopic Analysis of as Treated *Synechocystis* Cells

As treated *Synechocystis* cells were analyzed using a phase contrast microscope (Olympus BX53, Japan) and transmission electron microscope (TEM; FEI Tecnai G2 Twin, Netherlands). For all microscopic analysis, *Synechocystis* cells from mid-exponential growth phase were used. For phase contrast microscopy, 10 μl cell suspension was transferred to a glass slide and diluted with 50 μl of phosphate buffered saline PBS (pH 7.4). The slides were allowed to air dry, covered with cover-slip and then viewed at 100X magnification.

For surface analysis using TEM-energy dispersive X-ray spectroscopy (EDX), cells were washed with PBS once at 4000 rpm for 5 min. The washed cells were loaded onto the grid and viewed without negative/positive staining. The staining step was skipped as stains (i.e., phospotungstic acid or uranyl acetate) can cause intereference in As detection during EDX analysis. In case of unwashed cells, cells were diluted 10–15 times with PBS, viewed and analyzed at 80–120 kV. For intracellular analysis, the cells were fixed with 0.6% glutaraldehyde for 30 min and then post-fixed with 1% osmium tetraoxide. The cells were dehydrated with acetone series from 10 to 100% and then embedded with Spurr’s resin. The embedded cells were sectioned using an ultramicrotome (PowerTome, RMC Products, USA). The cell sections were viewed with TEM at 80 kV (for imaging) and 200 kV (for EDX analysis). EDX was done in TEM as well as in Scanning TEM (STEM) mode. Images were processed using Gatan’s Digital Micrograph software.

### Bioinformatics Analysis of *Synechocystis* PilA1-As Interaction

Phyre2 software (intensive mode; Kelley et al., 2015) was used to build the homology model of PilA1 (Kazusa Cyanobase-*sll*1694) as described earlier (Sure et al., 2015). The PilA1 structure was analyzed using Pymol software (©Schrodinger 2010, LLC). Docking studies were performed by using PatchDock software (Beta 1.3 version; Duhovny et al., 2002; Schneidman-Duhovny et al., 2005).

### Determination of Intracellular and Extracellular as Concentration

The extracellular and intracellular concentration of total As was determined from cells harvested at mid-exponential growth stage. Ten ml cell culture was taken and centrifuged at 6000 rpm for 4 min. The resultant supernatant was used for determination of extracellular concentration of As present in the medium. The remaining cell pellet was washed with Milli-Q once to remove surface bound As. The cells were resuspended again in ice-chilled phospate buffer [1 mM K_2_HPO_4_, 0.5 mM Ca(NO_3_)_2_, and 5 mM MES] for 10 min to remove the apoplastic As (Yin et al., 2011, 2012; Zhang et al., 2013). The cells were centrifuged again, resultant supernatant was discarded and the remaining cell pellet was oven dried. The dried cell pellet (4 mg each) was digested by adding 9 ml of [HNO_3_] and 3 ml of [HCL] and then subjected to microwave assisted digestion (MARS Microwave Digester, CEM Corporation, USA EPA Method 3015A). The digest was filtered using Whatman filter paper (Grade 41) and volume of the filtrate was made up to 25 ml using Milli-Q water (Bhattacharya and Pal, 2011). Flame atomic absorption spectroscopy (AAS; ICE 3500 AA System, Thermoscientific, UK) was used to determine the total concentration of As.

## Results and Discussion

### IC_50_ of Arsenic for *Synechocystis* Cells

**FIGURE 1:**
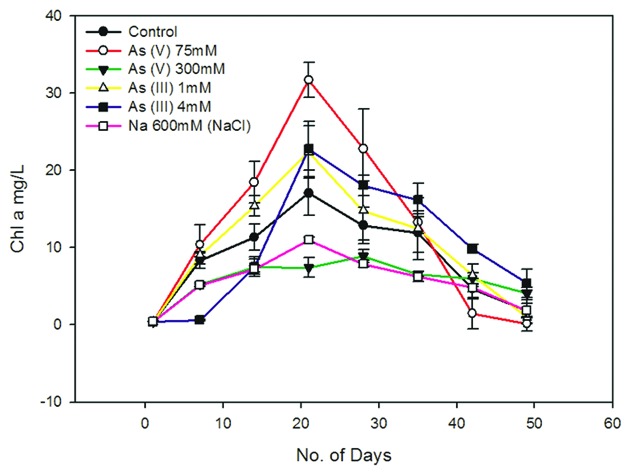
**Effect of arsenic and sodium ions (Na^+^) on *Synechocystis* cell growth.** 300 mM As (V) and 4 Mm As (III) causes around 40–50% of cell growth inhibition. 300 mM sodium arsenate (Na_2_HAsO_4_.7H_2_O) contains 600 mM of Na^+^. To study the effect of these Na^+^ on cell growth, cells were cultured with 600 mM NaCl and its effect on cell growth was studied along with As (V) treated and control cells. 300 mM sodium arsenate and 600 mM NaCl treated cells showed quite similar growth pattern, suggesting significant influence of Na^+^ on cell growth in arsenic treated cultures. Distinct increase in Chl a amount was observed in As treated *Synechocystis* cells after 7 days. 75 mM As (V) shows comparatively higher Chl a increase than 1 mM As (III). The data has been presented as mean ± standard deviation (*n* = 3).

In our preliminary analysis, a significant reduction in the cell growth was observed beyond 300 mM As (V) and 4 mM As (III). The Chl a and OD_750_ measurement of 14th day cultures (mid-exponential growth phase) treated with 300 mM As (V) and 4 mM As (III) showed that these As concentrations caused ~40–50% growth inhibition and thus these were used for further studies (**Figure 1**, Supplementary Figure S1). The high tolerance to As shown by the lab grown *Synechocystis* is comparable to the As hypertolerant *Bacillus* sp. strain DJ-1 [IC_50_ of 400 mM As (V) and 10 mM As (III)] which was isolated from an As contaminated site (Joshi et al., 2009). This suggests that the *Synechocystis* cells may have in built capacity to deal with high concentrations of As.

Sodium chloride concentrations ≥550 mM are known to induce salt stress in *Synechocystis* leading to significant changes in protein synthesis, ATP generation, etc. which in turn affects cell growth (Hagemann et al., 1991; Jeanjean et al., 1993). The 300 mM of sodium arsenate used in this study contains 600 mM of Na^+^ ions which is enough to cause salt stress to cells. Hence, we also studied whether the Na^+^ present in sodium arsenate can affect cell growth. We observed the growth curve of sodium arsenate and 600 mM NaCl treated cells to be quite similar (**Figure 1**, Supplementary Figure S1). This suggests that Na^+^ has a significant influence on cell growth in sodium arsenate treated cells. It also implied that cell inhibition in sodium arsenate treated cells, may be the sole effect of Na^+^, as same growth curve was observed with and without As. Thus, it seems that the actual tolerance level of As (V) for the *Synechocystis* cells might be much higher than that observed in our study. Future studies involving higher concentration of sodium arsenate should consider the effect of Na^+^ on *Synechocystis* cell physiology. Also to determine actual IC_50_ of As (V), cell tolerance studies need to be done against different As (V) compounds [e.g., arsenic pentasulfide (As_2_S_5_)] which will be future course of action.

Unlike As (V), the cells treated with 4 mM As (III) showed different growth behavior. No significant cell growth was observed up to 7 days during which the cells may be acclimatizing themselves to the toxic concentration of As (III). After 7 days, the cells showed gradual increase in growth till 21st day (**Figure 1**, Supplementary Figure S1). A previous report has shown that *Synechocystis* can tolerate up to 100 mM As (V) and 3 mM As (III) (Nagy et al., 2014). However, these results cannot be compared with our study, as authors of this paper neither mentioned it specifically as the maximum tolerable concentration nor provided any cell viability data at these concentrations. As (III) showed more toxicity (~100 times) to *Synechocystis* cells than As (V) (**Figure 1**, Supplementary Figure S1). Similar results have been observed in other cyanobacteria like *Microcystis aeruginosa*, *Nostoc muscorum, N. minutum*, and *Tolypothrix tenuis* (Ferrari et al., 2013; Wang et al., 2013).

*Synechocystis* cells showed increased Chl a production in the presence of non-inhibitory concentrations of As, i.e., at 75 mM As (V) and 1 mM As (III) (**Figure 1**). Increased Chl a production was also observed in 4 mM As (III) treated cells (**Figure 1**). There may be two reasons for observed increased Chl a production; (1) Photosynthetic organisms are known to produce higher Chl a in shading conditions to optimally use available light (Raghavendra, 2000; Beneragama and Goto, 2010). The possibility of increase in Chl a due to a shading effect was ruled out here by exposing all the cultures to the same light intensity, i.e., ~40–50 μmol photons m^-2^ s^-1^. (2) As may have caused growth stimulation in cells leading to increase in total Chl a level. Similar growth stimulatory effects of As have been observed in *Nostoc* species and *Chlorella vulgaris* (Ohki et al., 1999; Miyashita et al., 2012; Ferrari et al., 2013). We had carried out OD_750_ and cfu/ml analysis to confirm As induced growth stimulation in *Synechocystis* but there were ambiguities in obtained results and thus we could not confirm it.

### Phase Contrast Microscopy of Arsenic Treated *Synechocystis* Cells

**FIGURE 2:**
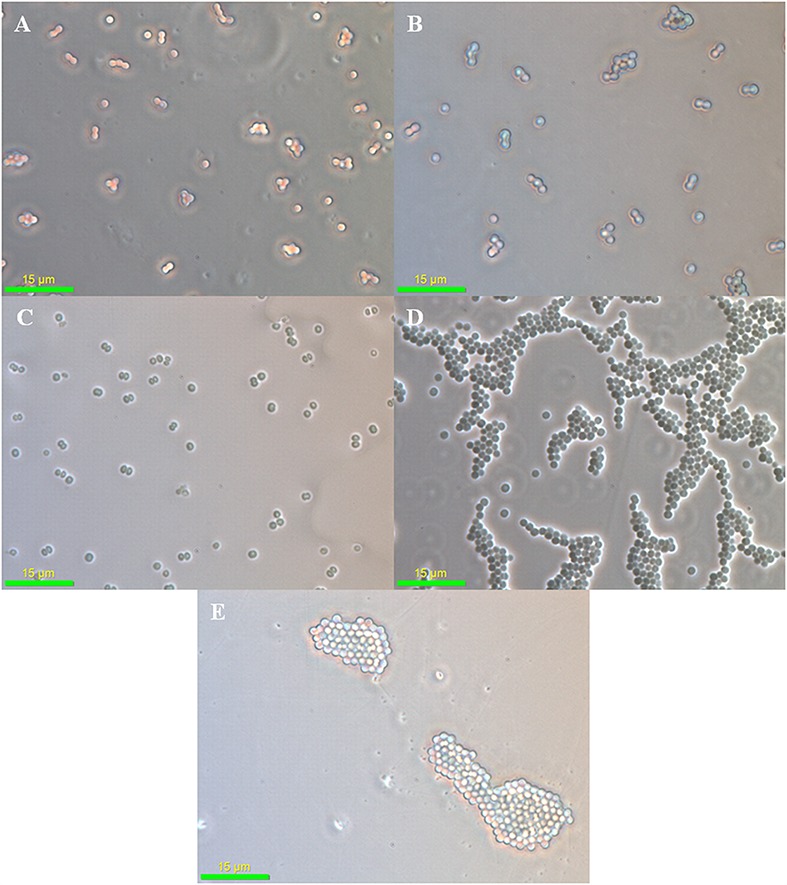
**Phase contrast microscopy analysis of arsenic treated *Synechocystis* cells.** No significant difference was observed in control **(A)**, 75 mM As (V) **(B)**, and 300 mM As (V) **(C)** treated cells. Strong aggregation of cells was observed at 1 mM **(D)** and 4 mM **(E)** As (III) concentration.

As (III) is approximately 100 times more toxic than As (V) for *Synechocystis* cells and this difference in As toxicity may force the cells to adapt different stress responses. When observed under the phase contrast microscope, no significant difference was observed in control and the As (V) treated cells (**Figures 2A–C**). However, the cell aggregation was observed in As (III) treated cells (**Figures 2D,E**). Such As (III) induced cell aggregation was also visible macroscopically (Supplementary Figure S2). As (III) is believed to cause oxidative stress in *Synechocystis* sp. (Hervaìs et al., 2012; Xue et al., 2014). Further, oxidative stress induced by microcystin (a toxin produced by *Microcystis*) is known to induce cell aggregation in *Synechocystis* (Li et al., 2009). Thus, cell aggregation observed here might be the result of oxidative stress induced by As (III). Such cell aggregation allows greater tolerance to toxic substances and stress conditions. Cell aggregation has previously been observed in *Nostoc muscorum* at 10,000 ppm As concentration (Ferrari et al., 2013).

### Arsenic Modulates Number and Length of TFP in *Synechocystis*

**FIGURE 3:**
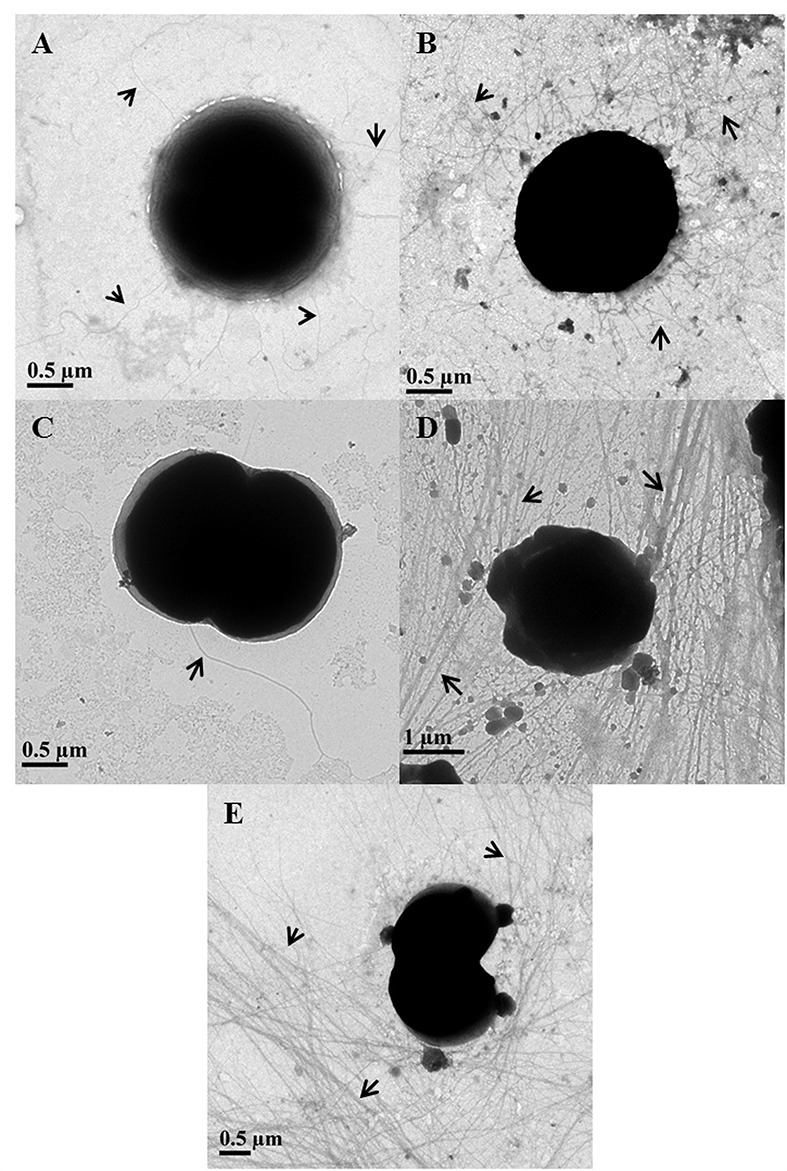
**Determination of piliation level in arsenic treated *Synechocystis* cells using transmission electron microscopy.** Control **(A)** and 75 mM As (V) **(B)** treated cells showed similar level of piliation while very few to almost no pili were observed in 300 mM As (V) treated cells **(C)**. Longer and high number of pili observed in cells treated with 1 mM **(D)** and 4 mM **(E)** concentration of As (III). Pili have been shown with black arrows.

In 75 mM As (V) treated cells, the piliation level appeared to be the same as in control cells (**Figures 3A,B**). Piliation level in 300 mM As (V) treated cells was quite low and pili were absent on most of the cells (**Figure 3C**). These results are in accordance with the previously published report where salt stress has been shown to suppress the expression of *pilA1*, which encodes structural protein of pili (Sergeyenko and Los, 2002).

The length of pili in control and As (V) treated cells usually varied from 2 to 5 μm and very few cells were observed to have long (≥10 μm) pili. Contrary to this, most of As (III) treated cells found to carry long (≥10 μm) and higher number of TFP than control and As (V) treated cells (**Figures 3D,E**). These results are consitent with phase contrast microscopy analysis as TFP in *Synechocystis* are believed to play a role in cell aggregation (Nakasugi et al., 2006; Galante et al., 2012) and it is also hypothesized that longer TFP help the cells to form large cell aggregates (Galante et al., 2012). Apart from cell aggregation, increased piliation may be involved in other functions, possibility of which was explored using bioinformatics studies.

### Bioinformatics Analysis of TFP-Arsenic Interactions in *Synechocystis*

The amino acids cysteine (C), aspartic acid (D), glutamic acid (E), and arginine (R) are known to be involved in binding with As (Santini and vanden Hoven, 2004). Proline (P) is known to bind various metals and also known to be involved in quenching of reactive oxygen species in plants (Matysik et al., 2002; Sharma and Dietz, 2006). *Synechocystis* PilA1, a monomer of TFP was found to be relatively rich in the above mentioned amino acids which hinted the possibility that *Synechocystis* TFP might be involved in extracellular As interaction (Supplementary Figure S3).

**FIGURE 4:**
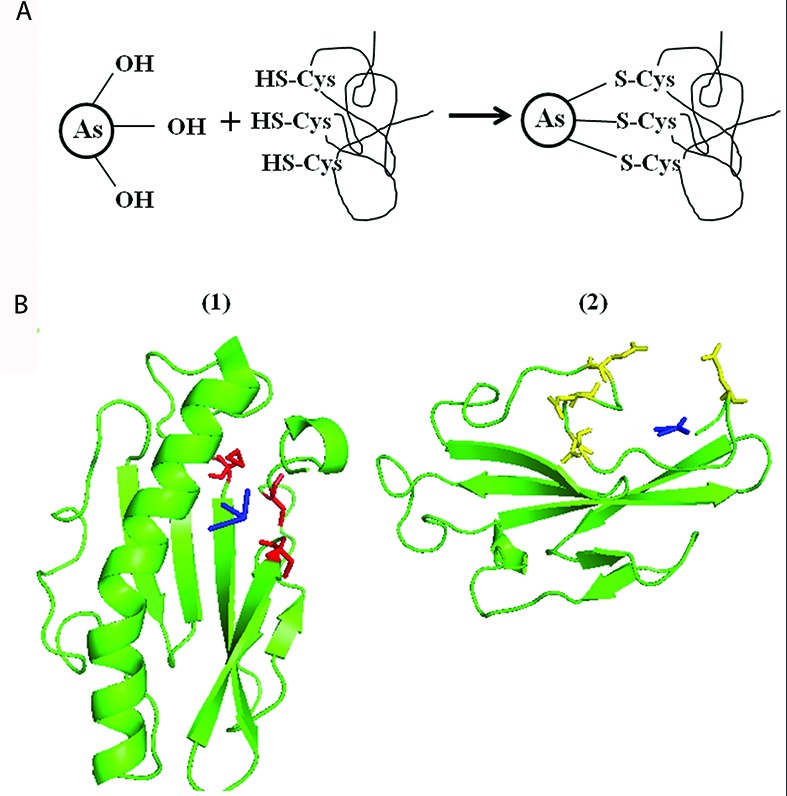
**Molecular docking of *Synechocystis* PilA and arsenic. (A)** Schematic representation of As (III) binding to three closely spaced sulfur containing amino acids (i.e., cysteine or methionine). **(B)** As (III) (shown in blue color) shows potential binding site at three -S containing amino acids (two cysteines and one methionine, shown in red color). (1) As (V) (shown in blue color) shows potential binding at C-terminal pocket rich in acidic amino acid (aspartic acid and glutamic acid, shown in yellow color). (2) For As (III) and As (V) docking, only area of interest of PilA1 has been shown.

To support our hypothesis, we bioinformatically analyzed the carboxyl-terminal structure of PilA1 which is predicted to be present on the outer surface of TFP (Craig et al., 2004; Sure et al., 2015). At the carboxyl-terminal, three sulfur containing amino acids [two C and one methionine (M)] are closely placed (Supplementary Figure S3). C is usually present in enzymes [e.g., As (V) reductase/oxidase] and proteins (e.g., repressor ArsR from *Escherichia coli*, metallothionein) involved in As reactions (Shen et al., 2013). As (III) has strong affinity for sulfhydryl group (-SH) present in C and binds to three residues of C as described in **Figure 4A** (Shen et al., 2013; Nagy et al., 2014). It is possible that As (III) binds in similar manner with the closely spaced three sulfur containing amino acids [two C (-SH) and one M (-S-CH_3_)] of PilA1 (Supplementary Figure S3). To evaluate this possibility, molecular docking was done using *Synechocystis* PilA1 as a receptor molecule and As (III) as a ligand. We observed that As (III) shows a potential binding site near the three sulfur containing amino acids, as discussed above (**Figure 4B1**). Both C and M can be involved in electron transfer processes and even act as a stepping stone in long range electron transfer (Wang et al., 2009; Sun et al., 2015). This suggests that PilA1 cannot only bind to the As (III), but may also be involved in its transformation. However, more elaborate studies are needed to confirm this hypothesis. R is predicted to be important in As reactions as it can interact directly with As and its intermediates (DeMel et al., 2004). R residues were observed to be adjacent to C residues in PilA1 (Supplementary Figure S3) suggesting their potential role in As interactions. The C-terminal of PilA1 was also found to be rich in acidic amino acids (D and E;Supplementary Figure S3). High content of D and E is indicative of greater ionic interactions and may be involved in As binding (Joshi et al., 2009). When As (V) was docked against the carboxyl terminal of PilA1, it showed a potential binding site in the acidic amino acid rich pocket (**Figure 4B2**).

The above observations suggests that *Synechocystis* TFP might be involved in extracellular interaction with As, and may have potential to interact with other metals also. Structure determination of PilA1 by experimental techniques (e.g., X-ray crystallography, Cryo-electron microscopy) will provide more detailed information about possible TFP-As interactions.

### *Synechocystis* TFP Interacts with Arsenic, Iron, and Manganese

**FIGURE 5:**
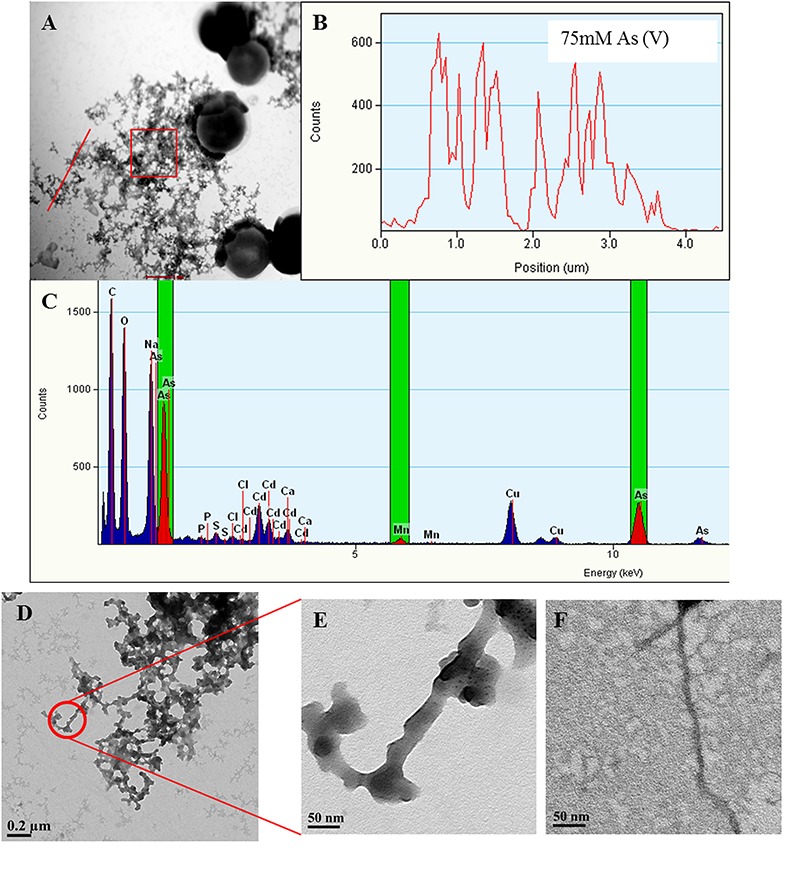
**Arsenic binds to *Synechocystis* TFP.** Representative images showing *Synechocystis* cells treated with 75 mM As (V) **(A)**. Line EDX spectra **(B)** and EDX of square area **(C)** (highlighted by red color in **A**) confirms the presence of As on putative TFP-As complexes. EDX spectrum **(C)** also shows small Mn peaks suggesting its possible presence on putative TFP. Line EDX data was collected from left to right direction (i.e., from bottom to top) image **(D,E)** are the high magnification images of putative TFP-As complexes (≥15 nm diameter). TFP from control cells (5–7 nm in diameter; **F)** at same magnification (X 29000) as that of putative TFP-As complex in image **(E)**.

**FIGURE 6:**
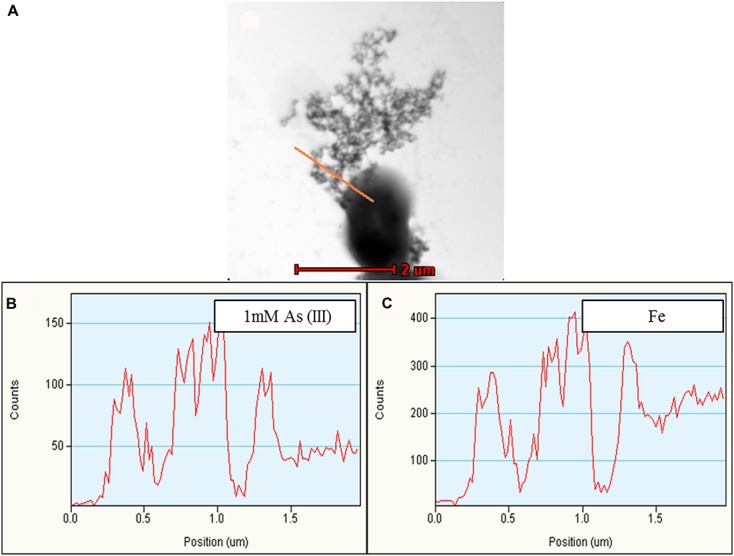
**Representative TEM-EDX analysis of *Synechocystis* cells treated with 1 mM As (III).** Putative TFP-As complexes were observed on *Synechocystis* cells **(A)**, which is confirmed by line EDX (highlighted by red line in **A**). **(B,C)** Fe was also observed along with As. Higher concentration of As was observed on putative TFP-As complexes than that of the cell. Line EDX data was collected from left to right direction (i.e., from top to bottom).

To confirm the results obtained from the bioinformatics analysis, TEM-EDX analysis of As treated *Synechocystis* cells was carried out. In agreement with *in silico* studies, putative TFP-As complexes were observed on *Synechocystis* cells for all tested concentrations of As (III) and (V), except in control cells (**Figures 5** and **6**, Supplementary Figures S4–S6). We have used term “putative TFP-As complexes” as our earlier study has confirmed that extracellular filaments produced by *Synechocystis* are proteinaceous in nature and are TFP (Sure et al., 2015).

Along with As, iron (Fe) and manganese (Mn) was also found to be deposited on TFP (**Figures 5** and **6**, Supplementary Figure S5). We have found extracellular deposits of Fe and Mn in control cells also (Supplementary Figure S4), albeit in a very few cells. In control and As treated cells, mainly four types of TFP were observed; (1) TFP with no As, Fe, or Mn deposition; (2) TFP bound to As only; (3) TFP bound to Fe, Mn, and As; (4) TFP bound to Fe or Mn or both. For case 2 and 4, it is possible that As, Fe, or Mn may be present on TFP but could not be detected due to a very low concentration. The line EDX shows that As, Fe, and Mn were colocalized on TFP (**Figure 6**; Supplementary Figure S5).

The deposition of As, Fe, and Mn on TFP seems to increase the total diameter of it (**Figures 5D–F**). The diameter of TFP associated with As and other metals was found to be around ≥15 nm (**Figure 5E**) while it was around 5–7 nm in control cells (**Figure 5F**). As discussed in the previous section, *Synechocystis* TFP are rich in amino acids which can bind with various metals. These electroconductive TFP, i.e., microbial nanowires (MNWs) may be helpful for microorganisms for extracellular interaction with toxic metals/metalloids. MNWs produced by *Geobacter sulfurreducens* and *Shewanella oneidensis* have been shown to be involved in electron transfer from cells to metals and found to be intertwined with Fe (III) oxides (Reguera et al., 2005; Gorby et al., 2006). MNWs of *Aeromonas hydrophila* have also shown a similar pattern when grown with ferric oxides (Castro et al., 2014). Thus, it may be possible that the conductive behavior of *Synechocystis* TFP and the presence of metal binding amino acids on it may have lead to deposition of As and other metals on it. The presence of any cytochrome like proteins on *Synechocystis* TFP surface and their potential role in As and other metal interactions needs to be explored to have complete understanding of such interactions.

To confirm whether TFP-As interaction in *Synechocystis* is Fe/Mn mediated or not, cells were grown in Fe and Mn deficient medium (i.e., Fe^-^Mn^-^ BG11). No extracellular deposition of Fe/Mn was observed in control and As treated cells grown in Fe^-^Mn^-^ BG11 (Supplementary Figures S7 and S8). This confirmed that Fe/Mn present in normal BG11 medium was the source of extracellular Fe/Mn deposits observed in *Synechocystis* cells (**Figures 5** and **6**, Supplementary Figures S4–S6).

When grown in Fe^-^Mn^-^ BG11, putative TFP-As complexes were observed on cells treated with 75 mM As (V) (Supplementary Figures S7C,D), while in case of 300 mM As (V) treated cells, As was observed in an extracellular dense, complex structure, the composition of which could not be confirmed (Supplementary Figures S7E–G). The formation of such dense, large extracellular As containing complexes was prominently observed in 300 mM As (V) treated cultures (Supplementary Figures S5 and S7E–G) and may be the result of high concentration of As present in medium. Putative TFP-As complexes was also observed in 1 and 4 mM As (III) treated cells which were grown in Fe^-^Mn^-^ BG11 (Supplementary Figure S8). Since putative TFP-As complexes was also observed in cells grown in Fe^-^Mn^-^ BG11 media, it can be concluded that TFP-As interaction is not mediated by Fe/Mn. Significantly higher deposition of As was observed on putative TFP than on cell surface (**Figure 6**, Supplementary Figures S5A,B, S7C,D, and S8E,F) which signifies potential of these structures in As accumulation from medium. Compared to control, reduced cell growth was observed in cells grown in Fe^-^Mn^-^ BG11 medium (Supplementary Figure S9) which is in accordance with earlier published studies (Odom et al., 1993; Salomon and Keren, 2011). Interestingly, for cells grown in Fe^-^Mn^-^ BG11 medium, presence of 75 mM As (V) was observed to ameliorate the absence of Fe/Mn in early stages of growth (Supplementary Figure S9).

There are two possibilities by which the binding of As to TFP may be important for cells. First, it may help to lessen the direct interaction with cell membrane and thereby minimizes damage to it. This may assist cells to have greater tolerance to As. Further, *Synechocystis* TFP have been shown to be electrically conductive in nature (Sure et al., 2015). Such electrically conductive pili, i.e., nanowires in *G. sulfurreducens* have previously been shown to act as a protective barrier against toxic metals like uranium (Cologgi et al., 2011) and it is possible that a similar mechanism may be operating here. The second possibility is that *Synechocystis* cells may use As as a photosynthetic electron source where electrically conductive TFP may be acting as conduit of electron transfer.

Bacteria have been known to deposit Fe and Mn on their extracellular structures (Ghiorse, 1984; Konhauser, 1997; Frankel and Bazylinski, 2003). *Synechocystis* requires significant amounts of Fe for electron transfer in photosynthetic as well as respiratory chain and different membrane proteins/transporters have been shown to be involved in its uptake (Jiang et al., 2012, 2014). *Synechocystis* does not produce siderophores and thus different mechanisms for Fe acquisition have been hypothesized (Kranzler et al., 2011). Here, we have observed that *Synechocystis* TFP are involved in Fe binding/precipitation from solution which later may be taken up by cells (Kranzler et al., 2011). These results support earlier research where *Synechocystis* PilA1 are shown to be involved in Fe acquisition (Lamb et al., 2014). Apart from Fe, Mn was also found to be deposited on TFP which is an important finding considering the fact that Mn is an essential part of water oxidizing centers of the cyanobacterial photosynthetic apparatus (Salomon and Keren, 2011).

Apart from electrically conductive TFP, other factors might be involved in extracellular precipitation of As, Fe, and Mn. For example, by oxygenic photosynthesis, cyanobacteria can increase oxygen concentration and pH, which in turn can assist in the precipitation of Fe and Mn (Nealson and Saffarini, 1994; Konhauser, 1997; Frankel and Bazylinski, 2003). The individual contribution of biotic and abiotic factors in extracellular precipitation of As and other metals needs to be explored further.

### Intracellular Analysis of *Synechocystis* Cells

*Synechocystis* is known to accumulate As intracellularly (Yin et al., 2011, 2012; Miyashita et al., 2012; Zhang et al., 2013). Effects of such intracellular accumulation of As on cell organelles have not been studied so far. Also it is not known whether As accumulates at any particular site in the cell. Apart from efflux of As, microorganisms are also known to store them in vacuoles (Mukhopadhyay et al., 2002). TEM-EDX analysis of As treated cell sections showed no distinct intracellular morphological differences in comparison to control cells (Supplementary Figures S10A–E). Increase in cell volume has been reported in bacteria due to intracellular accumulation of As (Pandey and Bhatt, 2015). No such increase in cell volume were observed in As treated *Synechocystis* cells (Supplementary Figures S10A–E) which may be the result of efflux of As by arsenite transporter. In TEM-EDX analysis, no As was detected intracellularly in As treated cells.

### Arsenic Concentration Determination Using Atomic Absorption Spectroscopy (AAS)

To complement the intracellular analysis of As in *Synechocystis* cells, extracellular and intracellular concentration of total As was determined using AAS. In case of 75 mM As (V) treated cells, ~90.65% (67.99 ± 1.7 mM) of total As was present extracellularly on 14th day while for 300 mM As (V), it was ~86.05% (258.15 ± 4.7 mM). For 1 mM As (III) treated cells, ~66% (0.66 ± 0.2 mM) As was present extracellularly on 14th day while for 4 mM As (III), it was ~75.75% (3.03 ± 0.4 mM).

The extracellular concentration of As (III) was considerably lower than As (V) which may be the result of intracellular uptake of As (III) by the cell. As (V) needs to compete with phosphate for transport into the cell while As (III) can be easily transported in the cells via aquaporins (Wang et al., 2013). This might be reason behind higher percentage of extracellular As (V) than As (III). Similar results have been observed in *M. aeruginosa* (Wang et al., 2013).

Intracellular As (V) and As (III) could not be detected as it may be present below the detection range (1 ppm) of flame AAS. To tackle As (V) toxicity, cells have developed a dedicated machinery which converts As (V) to As (III) [by As (V) reductase, ArsC] and selectively extrudes the latter out of the cell with the help of the specific transporter, ArsB (Li et al., 2003). The presence of As (III) transporter in *Synechocystis* might be the reason behind less intracellular presence of As (Yin et al., 2011; Miyashita et al., 2012; Zhang et al., 2013). High intracellular accumulation of As have been observed in bacteria lacking As (V) reductase and As (III) transporter (Joshi et al., 2009).

## Conclusion

The *Synechocystis*-As interaction studies done so far are mainly focused on enzymes and transporters involved in such interaction. Using bioinformatics tools, we showed that *Synechocystis* TFP have the potential to bind As and may be involved in As interaction due to its conductive nature. This hypothesis was further supported by TEM-EDX analysis where As was found to be precipitated on *Synechocystis* TFP. As also seems to modulate the length and number of *Synechocystis* TFP. Contrary to extracellular changes observed, As does not seem to induce any distinct intracellular morphological changes in *Synechocystis* cells. This study may stimulate further research on potential of microbial nanowires in arsenic immobilization and its potential implications in basic and applied aspects of microbiology.

## Author Contributions

SS conceived and developed the idea in consultation with MK, MLA, and AA; designed and performed experiments, conducted all bioinformatics analyses, analyzed data and wrote the manuscript with input from all co-authors, MLA supervised the work and gave critical comments on manuscript, AG provided significant technical assistance in TEM studies, PG performed TEM sample preparation, AA gave critical comments on manuscript, MK supervised all aspects of the work including planning and preparation of the manuscript and gave critical comments on manuscript. The manuscript is read and approved by all authors.

## Conflict of Interest Statement

The authors declare that the research was conducted in the absence of any commercial or financial relationships that could be construed as a potential conflict of interest.
