# Supplementary material for: Probing Synechocystis-Arsenic Interactions through Extracellular Nanowires
Source: Front Microbiol. 2016 Jul 19;7:1134. doi: 10.3389/fmicb.2016.01134 (PMC4949250; doi:10.3389/fmicb.2016.01134)
Supplement: Supplementary file 1 [file Data_Sheet_1.PDF]

SUPPORTING INFORMATION

Probing *Synechocystis*-arsenic interactions through extracellular nanowires

Running Title – *Synechocystis* nanowires-arsenic interaction studies

Authors

Sandeep Sure[1], M. Leigh Ackland[2], Aditya Gaur[1], Priyanka Gupta[1], Alok Adholeya[1] and Mandira Kochar[1*]

[1]TERI-Deakin Nanobiotechnology Centre, TERI Gram, The Energy and Resources Institute, Gual Pahari, Haryana, India, [2]Centre for Cellular & Molecular Biology, Deakin University, Melbourne, Victoria, Australia.

Corresponding Author

Email: mandira.malhotra@gmail.com or mandira.kochar@teri.res.in

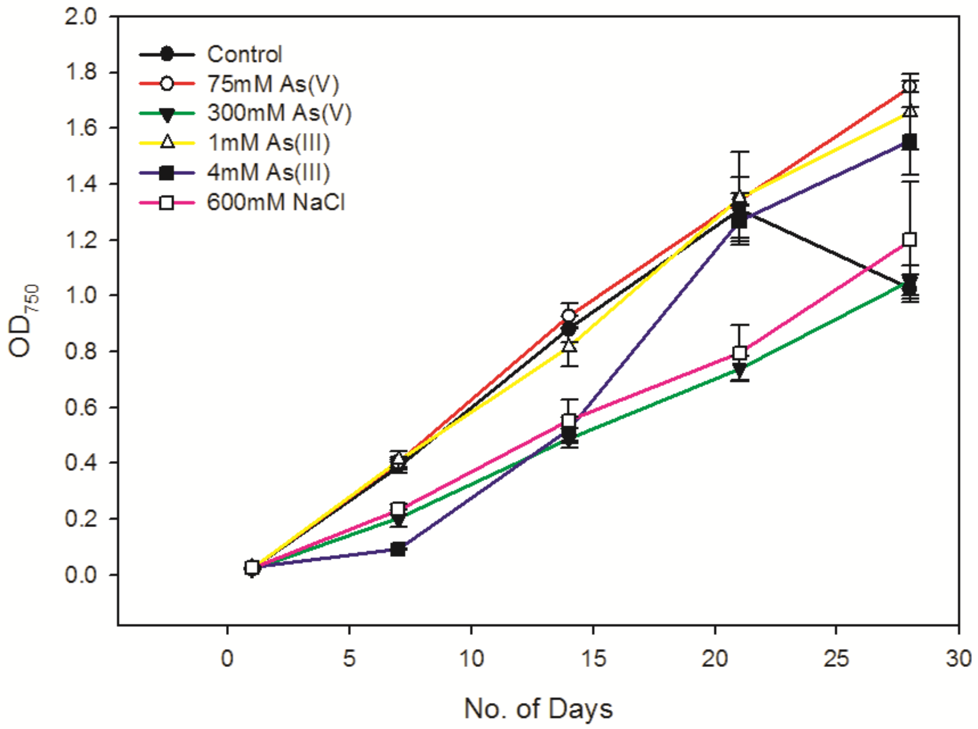

Figure S1. The growth behaviour of *Synechocystis* cells in presence of different concentrations of arsenic (As) and 600mM NaCl as determined by OD_{750} measurement.

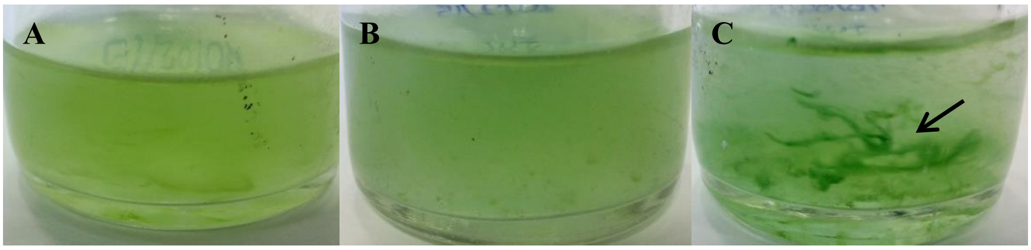

Figure S2. Arsenite causes cell aggregation in *Synechocystis*. Control (untreated) (A) and 1mM As (V) (B) treated cells show homogenous cell suspension while cells treated with 1mM As (III) (C) shows cell aggregation. Aggregated cells have been shown with black arrow.

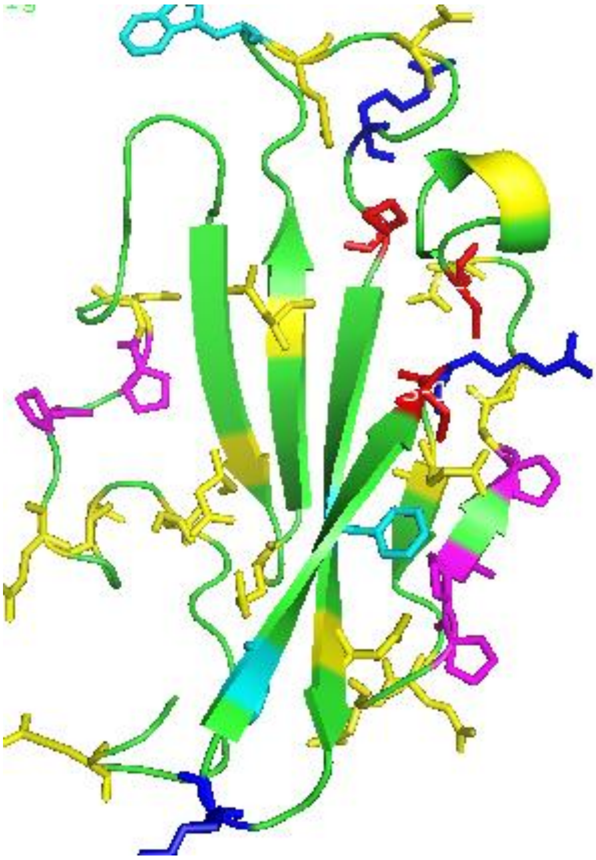

Figure S3. C-terminal of *Synechocystis* PilA showing closely placed cysteine/methionine residues (red coloured), arginine (blue coloured), aromatic amino acids (cyan coloured), aspartic and glutamic acid (yellow coloured) and proline (magenta coloured).

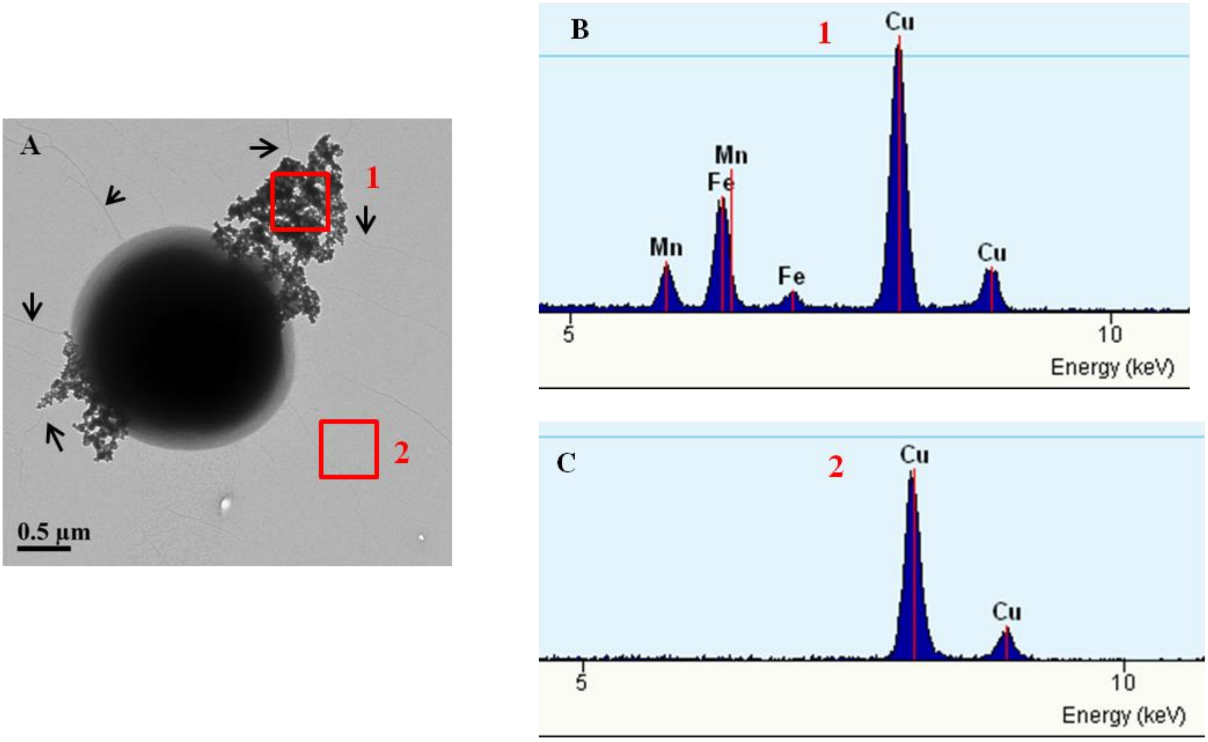

Figure S4. Representative TEM-EDX analysis of As untreated *Synechocystis* cell (control). Extracellular precipitates of Fe and Mn were observed (A) which was confirmed by TEM-EDX analysis (B, Spot 1). No Fe or Mn was observed in blank area (C, Spot 2). Red square shows the area where EDX was taken. *Synechocystis* TFP have been shown with black arrows.

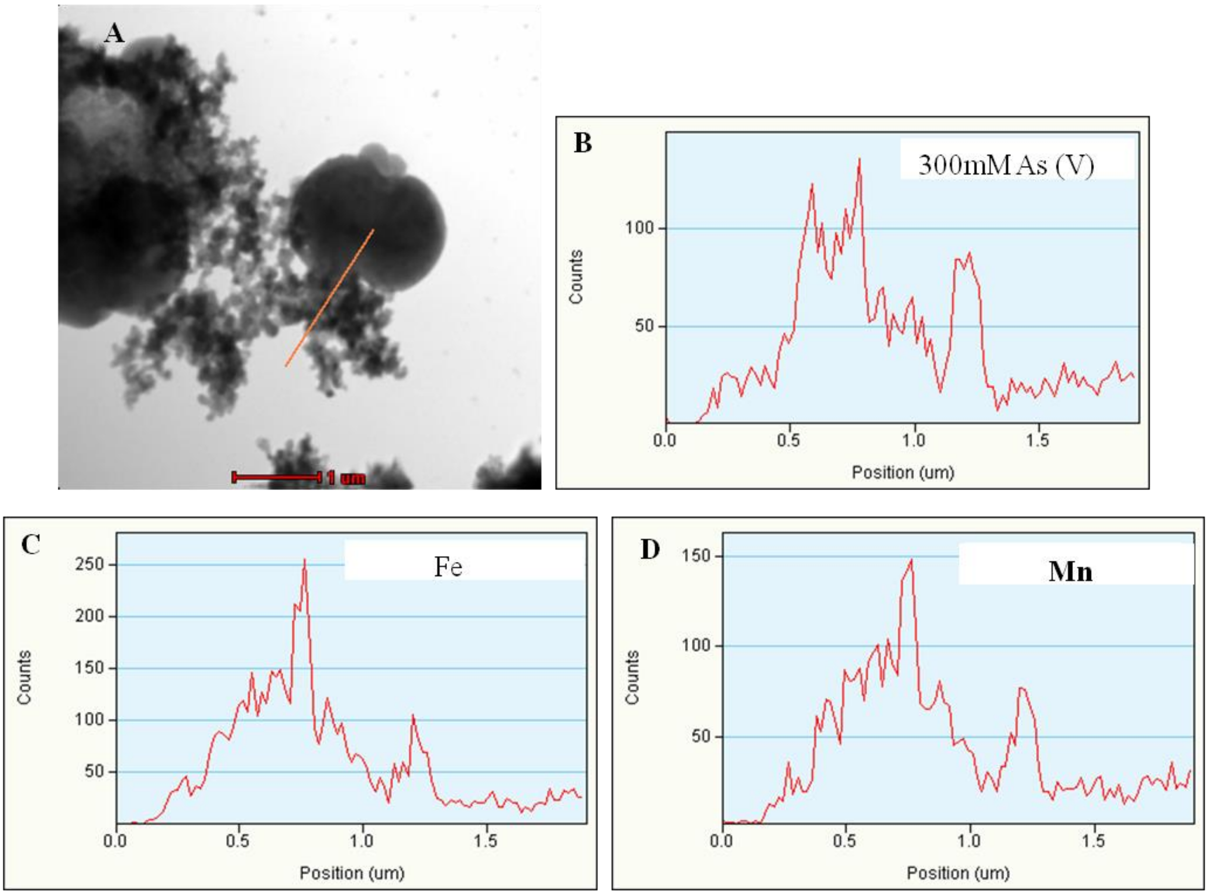

Figure S5. Representative TEM-EDX analysis of *Synechocystis* cells treated with 300mM As (V). Cells showing extracellular As precipitates (A) where presence of As, Fe and Mn was confirmed by line EDX (B-D) (highlighted by red line in A). Line EDX shows that As, Fe and Mn are colocalized. EDX data was collected from left to right direction (i.e. bottom to top). Higher concentration of As was observed on extracellular structures than that of the cell.

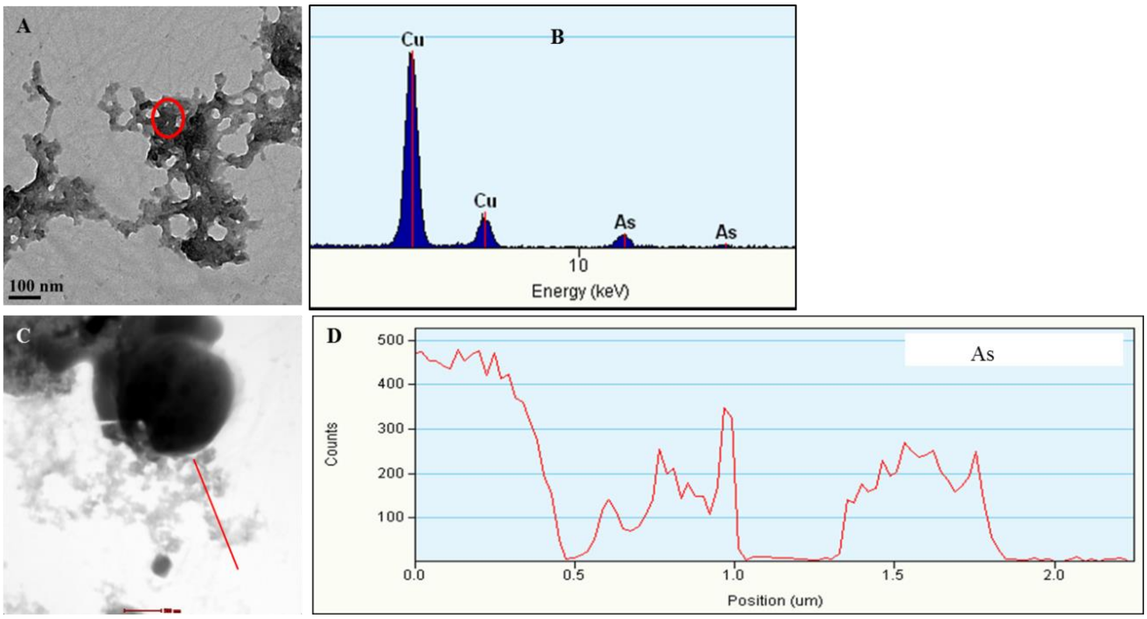

Figure S6. Representative TEM-EDX analysis of *Synechocystis* cell treated with 4mM As (III). Putative TFP-As complexes was observed on *Synechocystis* cells (A, C). The presence of As was confirmed by spot (B) and line EDX (highlighted by red line in C) (D). Line EDX data was collected from left to right direction (i.e. from top to bottom).

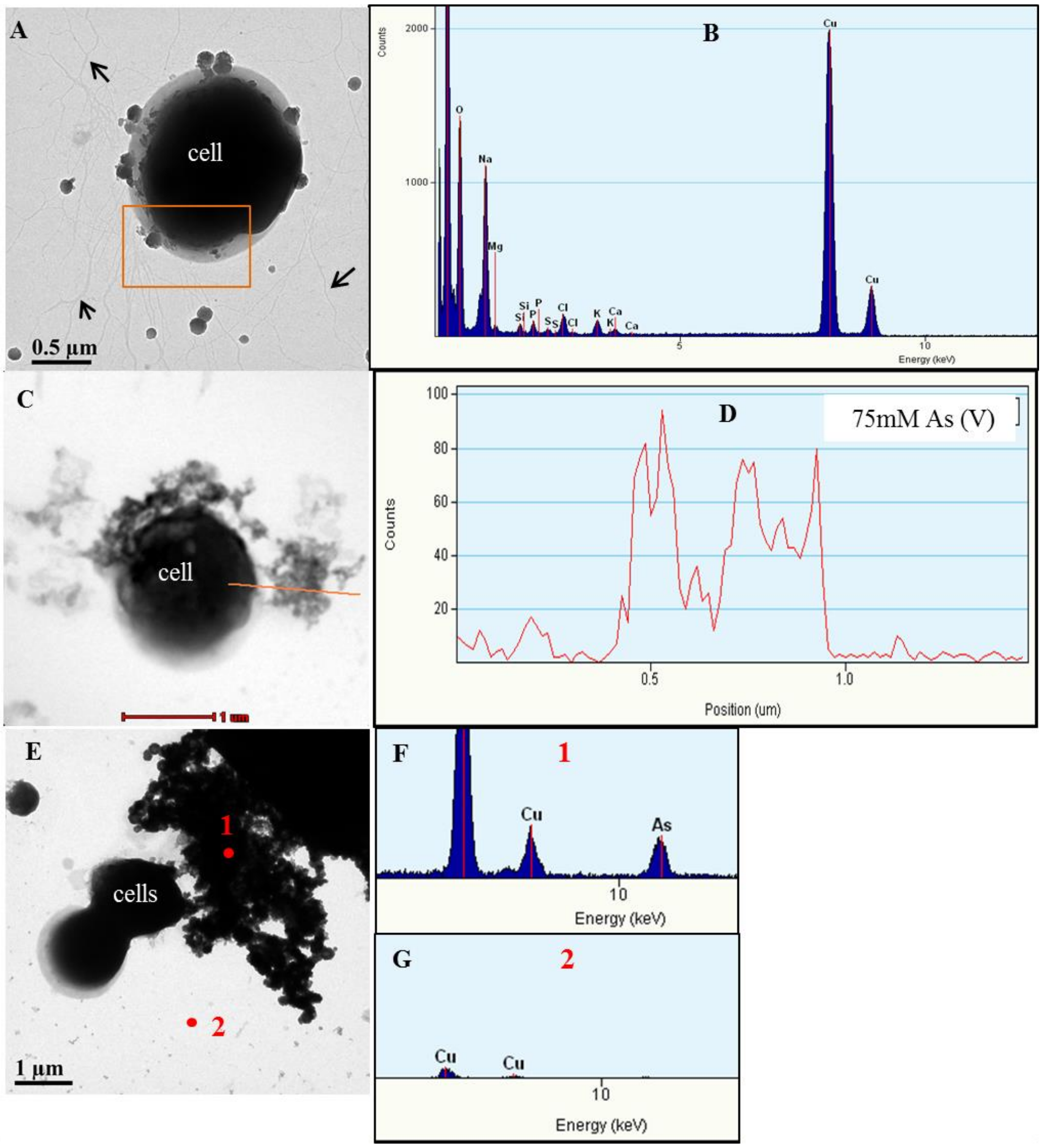

Figure S7: TEM-EDX analysis of *Synechocystis* cells grown in Fe⁻ Mn⁻ BG11 medium. Control cells (A) did not show the presence of As, Fe and Mn on cells or extracellular structures in EDX spectra (B). In image A, red square shows the area where EDX was taken. TFP have been shown with black arrows. 75mM As (V) treated cells (C) showed extracellular deposition of arsenic which confirmed by line EDX (highlighted by red line in C) (D). The extracellular deposition of As on putative TFP was significantly higher than on cells (see X-axis 0.5-10µm). Line EDX data was collected from left to right direction. 300mM As (V) treated cells (E) showed extracellular deposition of arsenic in large dense, complexes. The presence of As on these complexes was confirmed by EDX analysis of extracellular complex [Spot 1 (F)] and blank region [spot 2 (G)].

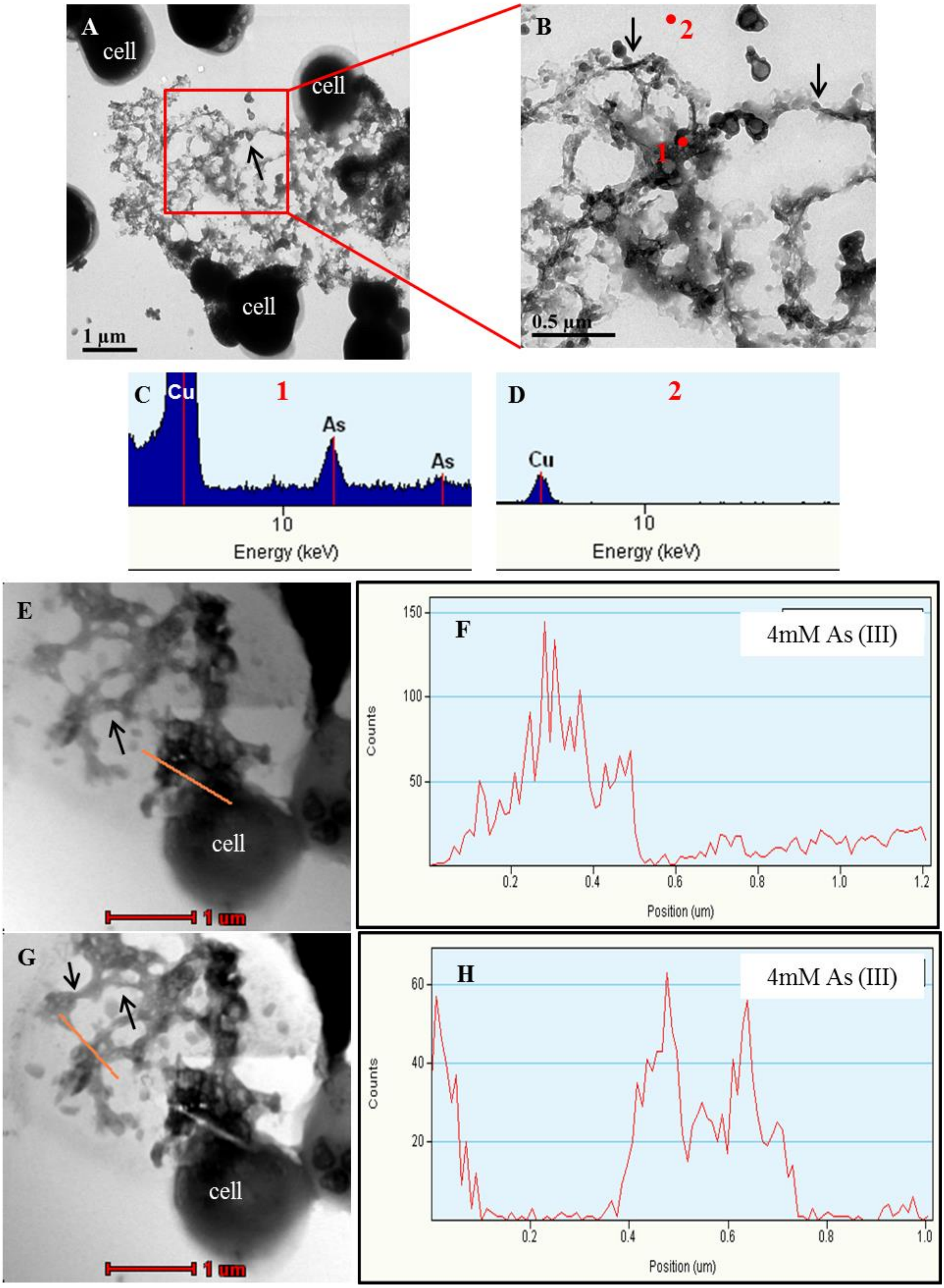
A
cell
cell
cell
1 µm
B
2
1
0.5 µm
C
Cu
1
As
As
10
Energy (keV)
D
2
Cu
10
Energy (keV)
E
cell
1 um
F
4mM As (III)
Counts
Position (um)
G
cell
1 um
H
4mM As (III)
Counts
Position (um)

Figure S8: TEM-EDX analysis of As (III) treated *Synechocystis* cells which were grown in Fe^{-} Mn^{-} BG11 medium. Putative TFP-As complexes was observed in 1mM As (III) treated cells (A-B). Image B shows the magnified view of putative TFP-As complexes. EDX analysis confirmed the presence of As on putative TFP (C, Spot 1) while no As was observed in adjacent blank area (D, Spot 2). In 4mM As (III) treated cells (E), As containing extracellular dense complexes were observed). Line EDX (highlighted by red line in E) showed higher deposition of As on extracellular complexes than on cell surface. Putative TFP-As complexes was also observed (G-H) along with extracellular As containing complexes. Putative TFP-As complexes have been shown with black arrow. Line EDX data was collected from left to right direction. .

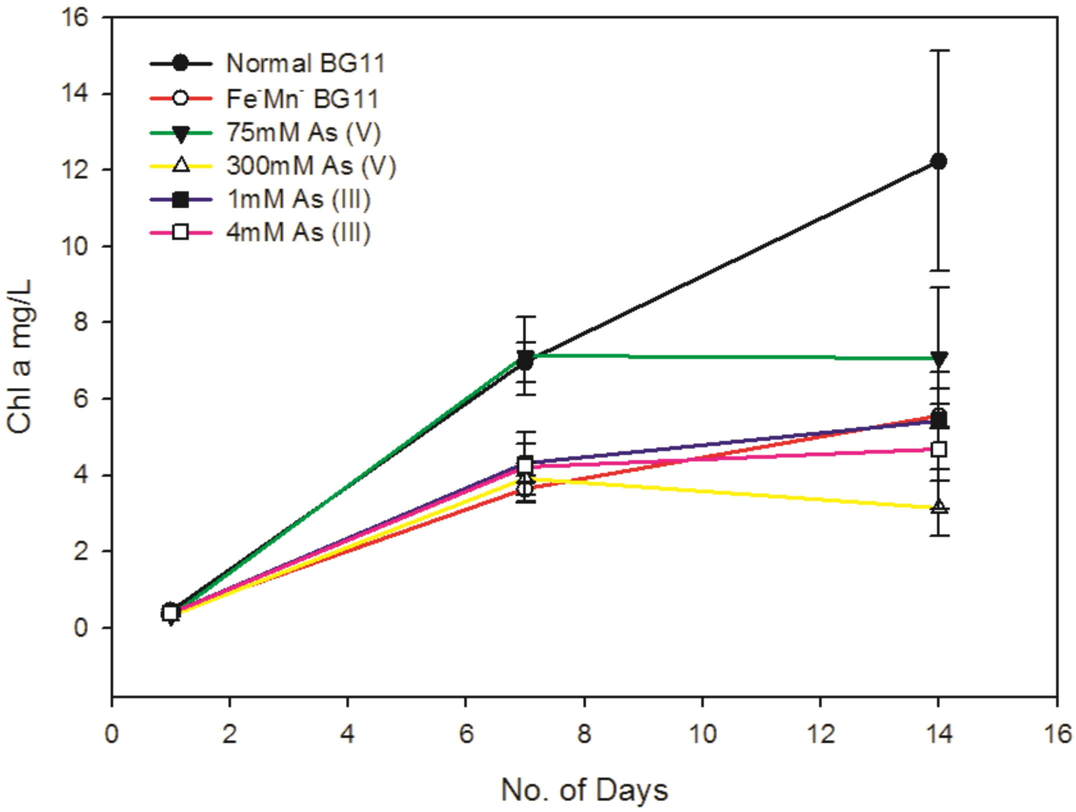

Figure S9. Growth behaviour of As treated and untreated *Synechocystis* cells grown in $Fe^{-}Mn^{-}$ BG11 medium. Arsenic treated and untreated cells grown in $Fe^{-}Mn^{-}$ BG11 medium show reduced cell growth compared to cells grown in normal BG11 medium, except that of 75Mm As(V) treated cells. Latter showed same growth behaviour as that of cells grown in normal BG11 medium till 7th day.

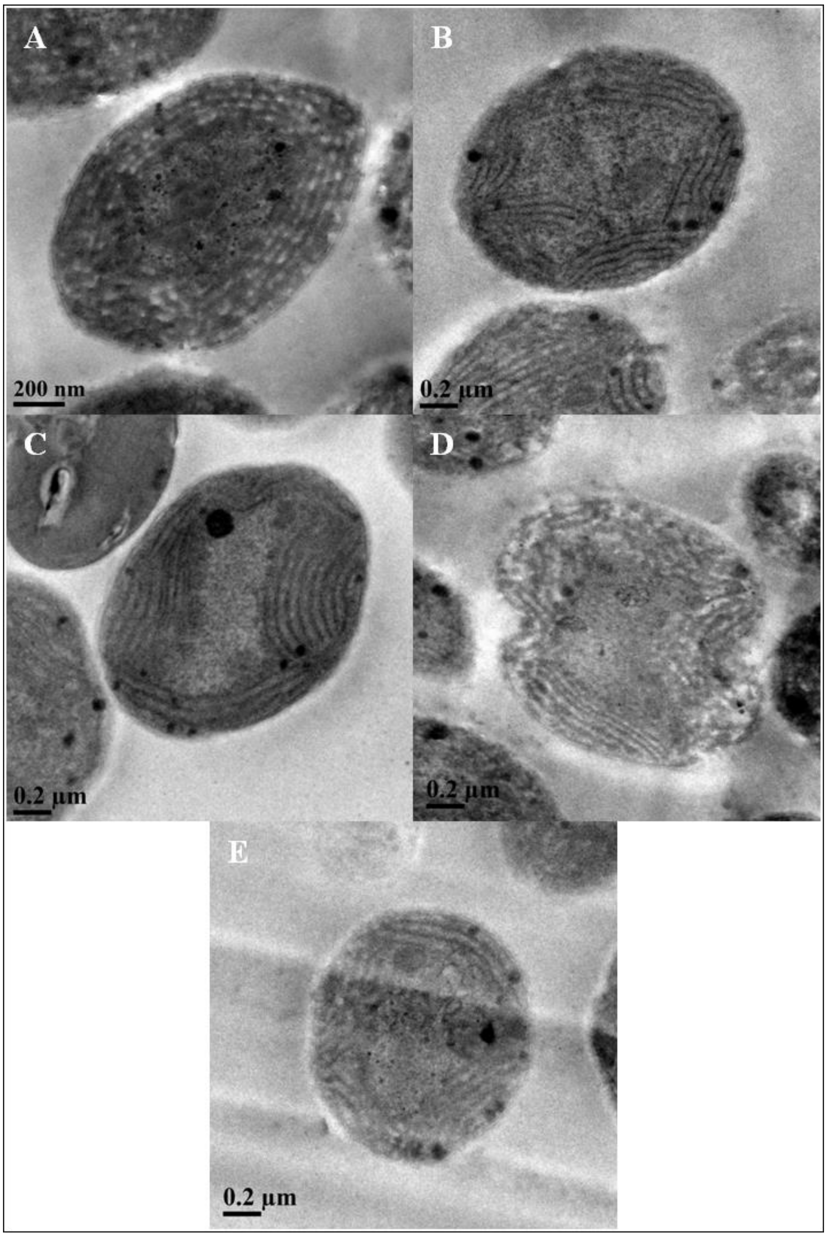

Figure S10. Intracellular analysis of arsenic treated *Synechocystis* cells using transmission electron microscopy. No significant morphological changes were observed in control (A) and As [As (V) 75mM (B) and 300mM (C); As (III) 1mM (D) and 4mM (E)] treated cells.
